# Daily exercises uptake and associated factors among Social Security and National Insurance Trust pensioners in the Greater Accra Region of Ghana

**DOI:** 10.1186/s41043-024-00655-8

**Published:** 2024-10-16

**Authors:** Myles Ongoh, Kwamina Abekah-Carter, Edmond A-iyeh, Williams Agyemang-Duah

**Affiliations:** 1 LEAP Management Secretariat, Ministry of Gender, Children and Social Protection, P.O. Box MB 471, Ministries-Accra, Ghana; 2 School of Social Work, Memorial University of Newfoundland, St. John’s, NL A1C 5S7 Canada; 3 Agaplesion-Diakonie Klinikum, Elise-Averdieck-Straße 17, 27356 Rotenburg, Germany; 4 Department of Geography and Planning, Queen’s University, Kingston, ON K7L 3N6 Canada

**Keywords:** Daily exercises uptake, Social Security and National Insurance Trust, Pensioners, Ghana

## Abstract

**Background:**

With a growing body of evidence highlighting the positive impact of regular physical activity or exercise on achieving healthy aging, it is important to gain insight into the factors influencing daily exercises uptake. However, to the best of our knowledge, no study has been focused on factors predicting daily exercises uptake among pensioners, who form a substantial portion of Ghana’s aging population. The goal of this preliminary study was to estimate the factors associated with daily exercises uptake among Social Security and National Insurance Trust (SSNIT) pensioners in Ghana.

**Methods:**

Data for this study came from a cross-sectional study on survival strategies and quality of life among SSNIT pensioners in the Greater Accra Region of Ghana. Cluster and stratified sampling techniques were used to recruit the study participants. The analytic sample was 410 participants. Multivariable binary logistic regressions were used to estimate factors associated with daily exercises uptake among the participants. The significance of the test was pegged at a *p*-value of 0.05 or less.

**Results:**

The results showed that 62% of the participants self-identified as male, 47.6% were aged between 60 and 64 years, 52.7% were employed in the public sector and 44.4% performed daily exercises. The results showed that those who were aged 60–64 years (AOR: 1.197, 95% CI: 1.019–1.405), aged 65–69 years (AOR:1.254, 95% CI: 1.071–1.468), who do not incur expenditure on their household in a month (AOR: 1.519, 95% CI: 1.127–2.046), earned less than GH¢260 (AOR: 1.221, 95% CI: 1.018–1.465), accessed/utilized herbalist medical services (AOR: 1.252, 95% CI: 1.129–1.388), very dissatisfied (AOR: 1.637, 95% CI: 1.242–2.157) and dissatisfied (AOR: 1.516, 95% CI: 1.212–1.896) with their sex life were more likely to undertake daily exercises and this was statistically significant. The results again demonstrated that participants who joined fitness club (AOR: 0.685, 95% CI: 0.614-0.764) and those who were very dissatisfied with their health services access/use (AOR: 0.598, 95% CI: 0.363-0.984) were less likely to undertake daily exercises and this was statistically significant.

**Conclusion:**

Findings of this study have provided important insights for policy makers and thus constitute a useful framework to help plan and shape future policies and programs on daily exercises uptake among pensioners in Ghana and other geographical contexts with similar cultural, demographic, and socio-economic characteristics.

## Introduction

Geographical territories worldwide are experiencing a shift in their demographics that is characterized by an aging population. It is estimated that the total global number of persons aged 60 years and above is about one billion, with expectations that by 2050, this number would increase by about 100% [1]. In Ghana, the population of persons aged 60 years and above accounts for over 6% of the national population [2], and projections made suggest that this proportion will rise to about 9.8% by 2050 [3]. The pensioner demographic, specifically those retiring from the formal workforce after reaching statutory retirement age, constitutes a significant part of this demographic shift [4, 5]. For instance, in Ghana, the mandatory retirement age for most formal sector workers is sixty years [6], aligning with the country’s age definition of an older person [7]. While the persistent increase in the number of older persons could be attributed to advancements in health care and living conditions [8, 9], the aging process is still associated with the probable onset of ailments and the loss of functional capabilities that are determined by diverse genetic, environmental, routine, and physical issues [10–12]. Moreover, retirement could be viewed as a social stress that can adversely affect one’s physical and mental health [13]. Consequently, as people transition to retirement, sustaining a healthy lifestyle becomes imperative for a rewarding and active life post-work life [14].

Physical exercise is an essential factor of a healthy lifestyle, and its importance to the aging population, including pensioners cannot be overemphasized. Exercise can be defined as regular, structured activity aimed at achieving suitable fitness outcomes, such as improving overall health and physical abilities [15, 16]. It is an essential physical activity routine that is useful to social adjustment, mental health, and cognitive function [17]. Some examples of exercise include walking, jogging, balance and stability exercise, cardiovascular exercise, flexibility exercise, bodyweight exercise, and strength training. In emphasizing the relevance of exercises, some experts note that physical inactivity does not only represent a loss of human potential, but it is also a risk factor for functional disability, poor health, and death [13, 15, 18]. Whereas physical activity may sometimes result in injuries and health complications [19], its positive effects outweigh the negatives [20]. Exercise contributes to slowing the progression of chronic conditions [21], and maintaining aerobic capacities [22], muscle mass, and strength [23]. Results from a study that examined the relationship between physical activity and incidence of coronary heart and cardiovascular illnesses among female older persons suggested that physical activity had a role in preventing these diseases among the study population [24].

A systematic review also found that physical activity improves cardiovascular outcomes among the aging population [25]. Furthermore, physical activity or exercise could reduce one’s risk of mobility issues [26], reduce the risk of falls [27], alleviate the fear of falling, improve balance sureness, quality of life, and physical performance [28, 29]. Physical activity or exercise also has a positive association with mental health. In Callow et al.’s [30] study on the advantages of physical activity on the mental wellbeing of older people in North America, it was revealed that older persons who were involved in greater levels of physical activity had a low risk of having depressive symptoms. Likewise, a study that discussed the effects of physical inactivity among older persons indicated that physically active older persons had a low risk of experiencing cognitive decline, dementia, Alzheimer’s disease, and depression [31]. Additionally, a study that assessed the impact of living alone on psychological distress among older persons in Ghana found that physical activity, including walking, dancing, sporting, and gardening could significantly reduce the negative link between living alone and emotional anguish [32]. Given the benefits of exercise, the Ministry of Health in Ghana recommends that older persons engage in 2.5 h of aerobic activity and two sessions of muscle-firming activities each week [33].

With a growing body of evidence shedding light on the positive impact of regular physical activity or exercise on achieving healthy aging, it is important to gain insight into the factors influencing daily exercises uptake. Some studies have reported that physical activity among older persons could be influenced by several variables, including sex [34] and marital status [35]. Furthermore, the availability of social support [36, 37], personal motivation factors [38–40], and the availability of requisite training facilitates could inform a person’s decision to exercise [39]. However, information on the daily exercise uptake among Ghana’s aging population and the factors that shape these behaviors remains scant as not much has been done to explore this issue. Only few studies, such as Balis et al. [33] have identified factors, such as peer influence, as well as suggestions from healthcare providers to be influential in older adults’ participation in physical activities in Ghana.

To the best of our knowledge, no study has been done to examine this topic among pensioners, who form a substantial portion of Ghana’s aging population. Accordingly, this study sought to supplement existing literature by investigating the daily exercises uptake and associated factors among Social Security and National Insurance Trust (SSNIT) pensioners in the Greater Accra Region of Ghana. Thus, the objective of this study was to explore factors influencing daily exercises uptake among pensioners in the Greater Accra Region of Ghana. This research is significant because by identifying the factors that influence daily exercises among this social group, healthcare officials, policymakers, and pensioners themselves could use this information to develop relevant interventions and strategies to improve overall health and quality of life during retirement.

## Data and methods

### Settings

**Fig. 1:**
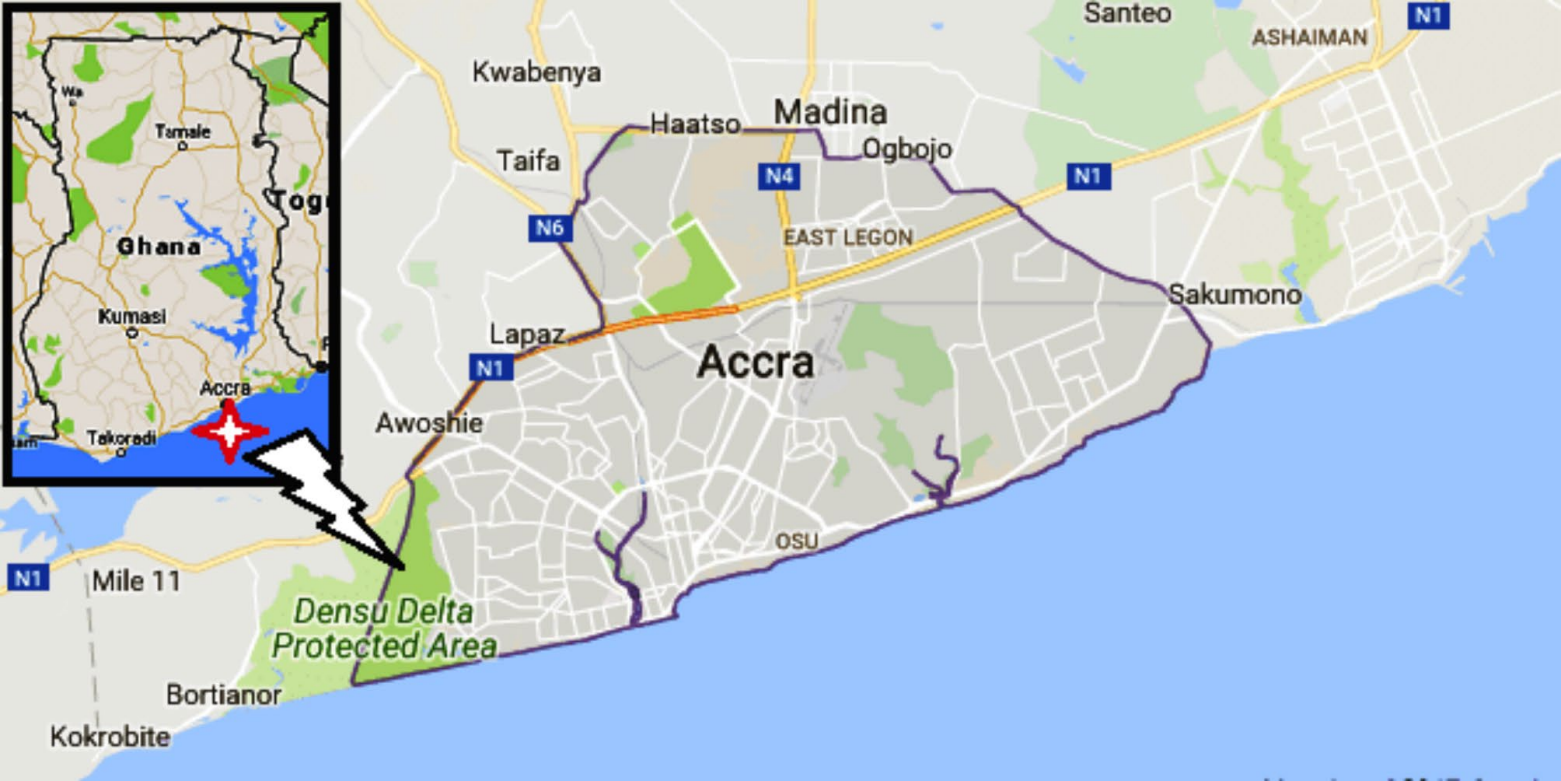
Map of Greater Accra in the context of Ghana

Located in the South-Eastern part of Ghana, Greater Accra Region shares boundaries with Eastern Region to the North, Volta Region to the East, Central Region to the West and Gulf of Guinea to the South with a total land area of 3, 245 km^2^ [41] as indicated in Fig. 1. Evidence suggests that the Greater Accra Region of Ghana has the highest pensioner population in Ghana making it an ideal location for this study.

### Research design

Data for this study came from a cross-sectional mixed methods study on survival strategies and quality of life among SSNIT pensioners in the Greater Accra Region of Ghana. This study specifically focused on an aspect of the larger cross-sectional mixed methods study, which looked at daily exercise uptake among SSNIT pensioners.

### Sampling procedure

In this study, we focused on SSNIT pensioners because SSNIT is the largest manager of pension funds in Ghana. Using Yamane [42] formula for sample size estimation, n$\:=\frac{N}{1+N{e}^{2}}$ [where n= the minimum sample size, z= the desired level of confidence level of 95% and the z-score corresponding to 95% confidence level=1.96, N= is population of pensioners in Greater Accra Region from the records of SSNIT in December 2016 was 49, 673 [43] and e= is the degree of precision which would be assumed to be 5%, hence *p*=0.05], we estimated a minimum sample size of 397. To cater for a non-response rate, we calculated a 10% non-response rate, resulting in a final sample size estimation of 437 pensioners. Participants were selected using stratified and cluster sampling techniques. The estimated sample size for this study was 437 pensioners. However, there were 27 missing values in some of the variables considered in this study. These were therefore excluded from the analysis. Hence, the analytic sample for this study was restricted to 410 participants.

### Data collection procedure and ethics

A structured questionnaire was used as the data collection instrument. It was designed in English and programmed on mobile devices with an electronic tool called Insyt; an easy, fast, robust, and flexible tool for collecting data. Institutional ethics approval was obtained from the College of Humanities at the University of Ghana, Legon (Ref: ECH 006/18–19). Both informed written and verbal consent were obtained from the participants. Detailed information on the methods, including the data collection procedure, has been reported elsewhere [5, 44].

### Measurement

In this study, our dependent variable was daily exercise uptake. Participants were asked, have you been undertaking daily exercises? The response was a dichotomous variable, that is, “no = 0” or ‘yes = 1. The independent variables were classified into demographic, socio-economic and lifestyle/health-related variables. Demographic variables were sex (0 = male, 1 = female), religion (0 = Christian, 1 = non-Christian), age (years) (0 = 60–64, 1 = 65–69, 2 = 70 or more), marital status (0 = consensual union, 1 = married, 2 = never married/Separated, 3 = widowed), household size (0 = 1–5, 1 = 6–10, 2 = above 10), household head (0 = no, 1 = yes) and years on retirement (0 = less than 5, 1 = 5–9, 2 = 10 or more). Socio-economic variables were expenditure on household (GH¢) (0 = less than 500, 1 = 500–999, 2 = 1000–1499, 3 = 1500 or more), education level (0 = none, 1 = primary/JHS/middle school, 2 = secondary, 3 = vocational/technical, 4 = tertiary), employment sector (0 = public, 1 = private), occupation (0 = administrative/managerial/clerical, 1 = civil/public service, 2 = entrepreneur/industrialist, 3 = production work, 4 = teacher/lecturer, 5 = other) and monthly income (GH¢) (0 = less than 260, 1 = 260–859, 2 = 860 or more 3 = did not disclose their income). Lifestyle/health-related factors included the use of herbalist medical services (0 = no, 1 = yes), joining of fitness club (0 = no, 1 = yes), satisfaction with health services access/use (0 = very dissatisfied, 1 = dissatisfied, 2 = neutral, 3 = satisfied 4 = very dissatisfied), satisfaction with sex life (0 = very dissatisfied, 1 = dissatisfied, 2 = neutral, 3 = satisfied 4 = very dissatisfied) and satisfaction with health status (0 = very dissatisfied, 1 = dissatisfied, 2 = neutral, 3 = satisfied 4 = very dissatisfied). Due to the several independent variables considered in the analysis, multicollinearlity analysis was performed as demonstrated in Table 1. The variance inflation factor (VIF) for all the independent variables was less than 5, showing no multicollinearity.

**Table 1:** Multicollinearlity analysis

Variables	Tolerance	VIF
Sex	0.633	1.579
Religion	0.951	1.052
Age (years)	0.485	2.061
Years on retirement	0.489	2.047
Marital status	0.713	1.403
Household Size	0.807	1.239
Household head	0.750	1.333
Expenditure on dependents (GH¢)	0.855	1.169
Education	0.871	1.147
Employment	0.863	1.159
Occupation	0.895	1.117
Monthly Income (GH¢)	0.868	1.152
Herbalist	0.887	1.127
Fitness Club	0.931	1.074
Satisfaction with health status	0.873	1.146
Satisfaction with sex life	0.880	1.136
Satisfaction with health services	0.827	1.209

### Analytical framework

In this study, both descriptive and inferential analytical frameworks, embedded in SPSS software version 25, were employed. Specifically, descriptive statistics such as frequency and percentage were used to determine the sample characteristics of the participants. Informed by the dichotomous dependent variable, multivariable binary logistic regression analysis as an inferential analytical framework was used to estimate the association between the dependent variable (daily exercises uptake) and independent variables (demographic, socio-economic and health-related/lifestyle factors). In applying the multivariable binary logistic regression, three models were fitted to determine factors associated with daily exercises uptake among the participants. More specifically, Model 1 comprised demographic variables. Model 2 consisted of demographic and socio-economic variables. Model 3 (final Model) captured demographic, socio-economic and lifestyle/health-related variables. The final model (3) thus serves as the result used for the discussion. Adjusted Odds Ratio (AOR) and Confidence Interval (CI) with *p*-value of 0.05 or less were reported as significant.

## Results

### Sample characteristics of the participants

Table 2 provides information on the sample characteristics of the participants. The analysis showed that 62% of the participants self-identified as male, 82.4% were Christians, 47.6% were aged between 60 and 64 years, 70.2% were married, 54.6% had a household size between 1 and 5 persons and 82.4% were household head. Also, 42.4% of the participants spent between GH¢500–999 on their household in a month and 42.4% had been on retirement for less than 5 years. Again, 35.6% of the participants had a primary/JHS/Middle school education, 52.7% were employed in the public sector and were engaged in production work (25.9%) and 55.1% earned between GH¢260–859 as a retirement benefit every month. Further, 23.9% of the participants used herbalist medical services, 19.8% joined fitness club, 25.9% were satisfied with health services access/use, 53.7% were satisfied with their health status and 48% were dissatisfied with their sex life. Lastly, 44.4% of the participants performed daily exercises.

**Table 2:** Sample characteristics of the participants

Variables	Responses	*N* = 410	Percent
Daily exercises	Yes	182	44.4
	No	228	55.6
Sex	Male	254	62.0
	Female	156	38.0
Religion	Christian	338	82.4
	Non-Christian	72	17.6
Age (years)	60–64	195	47.6
	65–69	138	33.7
	70 or more	77	18.7
Marital status	Consensual union	10	2.5
	Married	288	70.2
	Never married/Separated	37	9.0
	Widowed	75	18.3
Household size	1–5	224	54.6
	6–10	153	37.3
	Above 10	33	8.1
Household head	Yes	338	82.4
	No	72	17.6
Years on retirement	Less than 5 years	174	42.4
	5–9 years	144	35.1
	10 years or more	92	22.5
Expenditure on household (GH¢)	None	10	2.5
	Less than 500	126	30.7
	500–999	174	42.3
	1000–1499	41	10.0
	1500 or more	59	14.5
Education	None	3	0.7
	Primary/JHS/Middle School	146	35.6
	Secondary	53	12.9
	Vocational/Technical	64	15.6
	Tertiary	144	35.2
Employment sector	Public	216	52.7
	Private	194	47.3
Occupation	Administrative/Managerial/Clerifical	70	17.1
	Civil/Public Service	94	22.9
	Entrepreneur/Industrialist	20	4.9
	Production Work	106	25.9
	Teacher/Lecturer	77	18.8
	Other	43	10.5
Monthly income (GH¢)	Less than 260	47	11.5
	260–859	226	55.1
	860 or more	95	23.2
	Did not disclose	42	10.2
Use of herbalist medical services	Yes	98	23.9
	No	312	76.1
Joining of Fitness Club	Yes	81	19.8
	No	329	80.2
Satisfaction with health services access/use	very dissatisfied	4	1.0
	Dissatisfied	102	24.9
	Neutral	184	44.9
	Satisfied	106	25.9
	Very satisfied	14	3.3
Satisfaction with sex life	very dissatisfied	24	5.9
	Dissatisfied	197	48.0
	Neutral	104	25.4
	Satisfied	69	16.8
	very satisfied	16	3.9
Satisfaction with health status	very dissatisfied	7	1.7
	Dissatisfied	45	11.0
	Neutral	138	33.7
	Satisfied	220	53.6

### Main regression analysis

The factors associated with daily exercises uptake among the participants are reported in Table 3. In Model 1, the results showed that participants aged between 65 and 69 years were 1.309 times statistically significantly more likely to undertake daily exercises compared to those who were 70 years or more (Adjusted Odds Ratio [AOR]: 1.309, 95% CI: 1.098–1.560). In Model 2, when socio-economic variables were added to all variables in Model 1, the results demonstrated that those who were aged between 65 and 69 years were 1.286 times statistically significantly more probable to undertake daily exercises compared to those who were 70 years or over (AOR: 1.286, 95% CI: 1.078–1.535). Comparatively, the adjusted odds ratio for those aged 65–69 years reduced from 1.309 in Model 1 to 1.286 in Model 2. This implies that socio-economic variables slightly weaken the association between age and daily exercises uptake among the participants. In Model 2, the results showed that those who earned between GH¢260–859 in a month were 1.193 times statistically significantly more likely to undertake daily exercises (AOR: 1.193, 95%CI: 1.015–1.402). Again, the results indicated that those who did not incur expenditure on their household were 1.407 times statistically significantly more likely to undertake daily exercises compared to those who spent GH¢1,500 or more on their household in a month (AOR: 1.407, 95% CI: 1.016–1.949).

In the final model (3), the results showed that participants who were aged 60–64 years (AOR: 1.197, 95% CI: 1.019–1.405) and those who were aged 65–69 years (AOR:1.254, 95% CI: 1.071–1.468) were 1.197 and 1.254 times respectively, statistically significantly more likely to undertake daily exercises compared to those who were aged 70 years or over. The results showed that participants who did not incur expenditure on their household in a month were 1.519 times statistically significantly more likely to undertake daily exercises compared to those who incurred (AOR: 1.519, 95% CI: 1.127–2.046). We found that those who earned less than GH¢260 in a month were 1.221 times statistically significantly more likely to undertake daily exercises (AOR: 1.221, 95% CI: 1.018–1.465).

The results further revealed that participants who accessed/utilized herbalist medical services were 1.252 times statistically significantly more probable to undertake daily exercises compared to those who did not access medical services from herbalists (AOR: 1.252, 95% CI: 1.129–1.388). The results again provide evidence that participants who joined fitness club were 0.685 times less likely to undertake exercises compared to those who did not join fitness club, and this was statistically significant (AOR: 0.685, 95% CI: 0.614-0.764). Additionally, the results showed that participants who were very dissatisfied with their health services access/use were 0.598 times less likely to undertake daily exercises compared to those who were very satisfied with health services access/use, and this was statistically significant (AOR: 0.598, 95% CI: 0.363-0.984). The results also indicated that participants who were very dissatisfied (AOR: 1.637, 95% CI: 1.242–2.157) and dissatisfied (AOR: 1.516, 95% CI: 1.212–1.896) with their sex life were statistically significantly more likely to engage in daily exercise compared to those who were satisfied with their sex life, with odds ratios of 1.637 and 1.516, respectively. In summary, the results based on the final model have demonstrated that age, household expenditure, monthly income, use of medical services by herbalists, joining fitness club, satisfaction with sex life and satisfaction with health services access/use were statistically significantly associated with undertaking of daily exercises among the participants (see Table 3).

**Table 3:** Factors associated with daily exercises uptake among retired personnel in Ghana

	Model 1			Model 2			Model 3 (Final Model)		
	95% CI for AOR				95% CI for AOR			95% CI for AOR	
DEMOGRAPHIC	AOR	Lower	Upper	AOR	Lower	Upper	AOR	Lower	Upper
*Sex*									
Male	1.033	0.920	1.161	1.014	0.902	1.141	0.965	0.869	1.073
Female (ref)	1.00			1.00			1.00		
*Religion*									
Christian	1.082	0.956	1.224	1.059	0.933	1.202	1.038	0.927	1.163
Non-Christian (ref)	1.00			1.00			1.00		
*Age (years)*									
60–64	1.142	0.958	1.360	1.105	0.924	1.321	1.197*	*1.019*	*1.405*
65–69	1.309**	*1.098*	*1.560*	1.286**	*1.078*	*1.535*	1.254**	*1.071*	*1.468*
70 or more (ref)	1.00			1.00			1.00		
*Marital Status*									
Consensual union	0.745	0.533	1.042	0.785	0.562	1.098	0.859	0.635	1.162
Married	0.894	0.778	1.028	0.931	0.809	1.071	1.015	0.894	1.154
Never married/Separated	0.860	0.709	1.045	0.852	0.703	1.033	0.947	0.797	1.127
Widowed (ref)	1.00								
*Household Size*				1.00			1.00		
1–5	0.938	0.782	1.126	0.970	0.804	1.171	0.865	0.317	2.360
6–10	0.974	0.810	1.172	0.968	0.802	1.167	0.903	0.330	2.469
Above 10 (ref)	1.00			1.00			1.00		
*Household Head*									
Yes	0.884	0.768	1.017	0.908	0.789	1.045	0.978	0.863	1.110
No (ref)	1.00			1.00			1.00		
*Number of Years on Retirement*									
Less than 5 years	0.891	0.750	1.058	0.935	0.789	1.109	0.890	0.764	1.036
5–9 years	0.897	0.759	1.059	0.933	0.791	1.101	0.885	0.763	1.026
10 years or more (ref)	1.00			1.00			1.00		
SOCIO-ECONOMIC FACTORS									
*Education*									
None				0.906	0.520	1.578	0.787	0.480	1.291
Primary/JHS/Middle School				1.022	0.902	1.158	1.034	0.924	1.156
Secondary				1.087	0.923	1.281	1.038	0.896	1.203
Vocational/Technical				1.004	0.865	1.165	1.025	0.898	1.170
Tertiary (ref)				1.00			1.00		
*Employment Sector*									
Public				1.061	0.913	1.232	0.977	0.853	1.120
Private (ref)				1.00					
*Occupation*									
Administrative/Managerial/Clerical				0.951	0.789	1.147	0.966	0.816	1.144
Civil/Public Service				0.939	0.780	1.130	0.976	0.825	1.155
Entrepreneur/Industrialist				0.930	0.712	1.215	0.865	0.681	1.099
Production Work				1.057	0.876	1.276	0.995	0.841	1.177
Teacher/Lecturer				0.837	0.687	1.019	0.885	0.742	1.057
Other (ref)				1.00			1.00		
*Monthly Income (*GH¢)*)*									
Less than 260				1.199	0.977	1.471	1.221*	*1.018*	*1.465*
260–859				1.193*	*1.015*	*1.402*	1.096	0.946	1.269
860 or more				1.005	0.835	1.210	1.015	0.861	1.197
Did not disclose their income (ref)				1.00			1.00		
*Expenditure on Household (*GH¢)									
None				1.407*	*1.016*	*1.949*	1.519**	*1.127*	*2.046*
Less than 500				1.050	0.906	1.218	1.062	0.928	1.215
500–999				1.077	0.885	1.310	1.117	0.937	1.331
1000–1499				1.040	0.890	1.215	1.033	0.897	1.189
1500 or more (ref)				1.00			1.00		
LIFESTYLE/HEALTH-RELATED									
*Use of Herbalist Medical Care*									
Yes							1.252***	*1.129*	*1.388*
No (Ref)							1.00		
*Joining of Fitness Club*									
Yes							0.685***	*0.614*	*0.764*
No (ref)							1.00		
*Satisfaction with Health Services*									
very dissatisfied							0.598*	*0.363*	*0.984*
Dissatisfied							1.085	0.847	1.390
Neutral							1.059	0.832	1.349
Satisfied							1.231	0.964	1.572
Very satisfied (ref)							1.00		
*Satisfaction with Sex Life*									
very dissatisfied							1.637***	*1.242*	*2.157*
Dissatisfied							1.516***	*1.212*	*1.896*
Neutral							1.552***	*1.237*	*1.947*
Satisfied							1.248	0.987	1.578
very satisfied (ref)							1.00		
*Satisfaction with Health Status*									
very dissatisfied							0.976	0.702	1.355
Dissatisfied							1.004	0.869	1.160
Neutral							1.026	0.932	1.129
Satisfied (ref)							1.00		

NB: Italic and asterisks values and indicate significance of the test

*Test is significant at the 0.05 level

** Test is significant at the 0.01 level

*** Test is significant at the 0.001 level

## Discussion

This study explored the factors that influence daily exercises uptake among pensioners in the Greater Accra Region of Ghana. The findings supplement extant literature on this research area and highlight the need for holistic approaches that consider socio-demographic and lifestyle/behavioural factors when formulating and implementing policies and programs intended to promote healthy lifestyles among older persons during retirement. The findings of this research suggest that pensioners within the age bracket of 65 and 69 years are more likely to undertake daily exercises compared to those who are 70 years or above. This finding corroborates the results of a systematic review, which reported that various forms of physical activities progressively decrease with age among older persons [45]. It is also partly consistent with the findings of Ishikawa-Takata et al. [46], which indicated that physical activity was significantly higher among older persons aged 65–74 years compared to those aged 75 years and above.

Some plausible reasons could be attributed to the decline of exercise uptake among older persons as they age. For instance, Debpuur et al.’s [47] research on the self-reported health and functional limitations among older persons in Ghana revealed that the reportage of poor health, which also hindered their functional capability, increased with age among both older women and older men. Moreover, some older persons assume poor health as an inevitable result of aging, and thus, they are not motivated to adopt healthy behaviours like exercise [48]. Furthermore, Rai et al.’s [49] study, which explored physical activity among retired older persons also found that even with heightened demands during their working days, some retirees believed that the nature and structure of their work provided a framework that facilitated the incorporation of exercise into regular routines, thereby preventing procrastination. However, after retiring, some retirees faced challenges in adjusting to a post-retirement routine; a factor they acknowledged as crucial for engaging in physical activity [49]. Therefore, promoting and providing support for the establishment of post-retirement routines among pensioners could be useful in sustaining regular exercise behaviours.

Nonetheless, after accounting for socio-demographic variables, the adjusted odds ratio for the association between being aged 65–69 and daily exercises decreased slightly, underscoring the importance of considering socio-economic factors in understanding and promoting exercises uptakes and general healthy behaviours among older persons. The socio-economic factors that recorded a significant association included earning between GH¢260–859 and earning less than GH¢260 every month. This finding is in line with Doubova et al.’s [50] research, which reported that older adults with stable income were more likely to engage in physical activities, such as exercises. Nonetheless, an interesting observation in this study’s finding is that the adjusted odds ratio for engaging in daily exercises generally decreased as the monthly income increased. It is not too clear what accounts for this as extant evidence [e.g. 51] suggests that sedentary time decreases with the increase of income. Probably, the pensioners earning higher monthly income had either more sedentary behaviours or had alternative forms of healthy lifestyles other than exercises. Conducting research to explore the motivation for undertaking exercise among older persons or pensioners earning lower and high incomes could be essential to increasing the depth of knowledge and providing clarity to this issue. The study also found that pensioners who incurred no household expenditure were more likely to engage in daily exercises. This suggests that such pensioners may be experiencing lower financial burdens, creating a conducive environment for prioritizing healthy habits, including undertaking daily exercises. Thus, it would be useful if strategies geared towards improving daily exercises uptake among pensioners or older persons include enhancing financial access to physical activity opportunities [52].

The findings also show a significant potential association between herbalist medical services and daily exercises uptakes. This supports the findings of existing studies that have demonstrated the importance of herbal medicine for enhancing exercise performance. For instance, a study conducted by Tao and colleagues [53] in China found that older persons who drank herbal tea involved themselves in regular physical exercises. Additionally, a study conducted in Korea found that the consumption of a traditional herbal mixture (known as HemoHim) increased exercise performance [54]. There is also the likelihood that pensioners who seek herbal care may have an all-inclusive perspective towards health and wellness, viewing both herbal remedies and physical activity as complementary elements of a healthy lifestyle. Conducting qualitative studies could provide an in-depth insight into this crucial association. The findings of this study further suggest a potential relationship between dissatisfaction with health services and a decreased likelihood of daily exercise uptake. It could be inferred from this that barriers to healthcare access could negatively influence individuals’ motivation for physical activity or exercise.

Interestingly, this study found that pensioners who joined fitness clubs were less likely to exercise daily. For some older persons, exercising in groups does not only motivate them to continue this important routine [55], but it has also been found to be effective in reducing risks of falls, functional decline, and depressive symptoms compared to exercising alone [56]. In addition to the health benefits associated with participating in the activities of fitness clubs, engaging in daily exercises could offer more health benefits to pensioners. Therefore, most pensioners who join fitness clubs are likely to miss these additional health benefits associated with daily exercise because of their lower odds of engagement in daily exercise. We are of the view that engaging in daily exercise is expected to strengthen the health of pensioners. This finding supports the need to encourage pensioners who joined fitness clubs to exercise daily. The findings also indicated that pensioners who were dissatisfied with their sex life were more likely to participate in daily exercises. These pensioners may be motivated to undertake daily exercises due to the belief that physical fitness could lead to the improvement of sexual satisfaction [57]. Therefore, sensitization campaigns that focus on the relevance of exercises, as well as specific exercise routines that enhance sexual satisfaction should be promoted. However, it is also possible for the finding to suggest a potential compensatory behavior, where engaging in regular exercise serves as a means for emotional regulation or distraction from sexual dissatisfaction. Seeking emotional relief through regular exercises emphasizes an intricate interplay between psychological and lifestyle factors. It would be useful if research is conducted among the aging population to offer a nuanced understanding on this issue.

Given the nature of this study, it is important to acknowledge its strength and limitations. The strength of this study is that it remains the first study to be carried out among pensioners in Ghana. It has thus contributed empirically to knowledge by highlighting the specific demographic, socio-economic and lifestyle/health-related factors predicting daily exercises uptake among pensioners in Ghana. Despite this, we emphasize that one major limitation of this study was the cross-sectional nature of the study which did not allow causal associations to be established between the dependent (daily exercises uptake) and independent variables (demographic, socio-economic and lifestyle/health-related variable). We further acknowledge that in terms of the measurement of the daily exercise uptake, this study did not clearly highlight the specific forms and durations of daily exercises uptake by the participants. Another possible limitation of this study is that it was conducted in one region (Greater Accra) in Ghana. Due to entirely different situations in the various areas of Ghana, the results of this study may not reflect the perspective of SSNIT pensioners in the other regions of Ghana. The above limitations offer opportunities for future studies to employ longitudinal data to analyze daily exercise uptake among the participants. Building on the findings of this preliminary study in the Ghanaian context, future research could determine the specific forms and durations of daily exercise uptake among Ghanaian pensioners across all regions in Ghana.

## Conclusion

This study examined factors associated with daily exercises uptake among SSNIT pensioners in Ghana. The study found that age, household expenditure, monthly income, use of medical services by herbalists, joining fitness club, satisfaction with sex life and satisfaction with health services access/use were statistically significantly associated with uptake of daily exercises among the participants. The findings of this study provide valuable insights for policymakers and offer a useful framework for planning and shaping future policies and programs aimed at increasing daily exercises uptake among pensioners in Ghana, and in other geographical contexts with similar cultural, demographic, and socio-economic characteristics. These findings further suggest the need for holistic approaches that consider socio-demographic factors and lifestyle/health-related factors when formulating and implementing policies and programs intended to promote healthy lifestyles among older persons during retirement.

## Data Availability

The datasets used and/or analysed during the current study are available from the corresponding author on reasonable request.

## Abbreviations

VIF: Variance Inflation Factor
SSNIT: Social Security and National Insurance Trust
SPSS: Statistical Package for Social Sciences
AOR: Adjusted Odds Ratio
CI: Confidence Interval
GH¢: Ghana Cedis
JHS: Junior High School
